# Microstructure and Strengthening Model of Cu–Fe In-Situ Composites

**DOI:** 10.3390/ma13163464

**Published:** 2020-08-06

**Authors:** Keming Liu, Xiaochun Sheng, Qingpeng Li, Mengcheng Zhang, Ningle Han, Guangyu He, Jin Zou, Wei Chen, Andrej Atrens

**Affiliations:** 1 Jiangxi Key Laboratory for Precision Actuation and Control, Nanchang Institute of Technology, Nanchang 330099, China; 2016984620@nit.edu.cn (X.S.); 13155981921@163.com (M.Z.); h18338811123@163.com (N.H.); hgy18839125513@163.com (G.H.); 2 Nanchang Electric Power Supply Company, State Grid, Nanchang 330012, China; liqingpeng@jx.sgcc.com.cn; 3 Institute of Applied Physics, Jiangxi Academy of Sciences, Nanchang 330096, China; zoujin@jxas.ac.cn (J.Z.); chenw@alum.imr.ac.cn (W.C.); 4 Centre for Advanced Materials Processing and Manufacturing, The University of Queensland, Brisbane, QLD 4072, Australia; andrejs.atrens@uq.edu.au

**Keywords:** microstructure, strength, evolution, model, in-situ composite, Cu–Fe

## Abstract

The tensile strength evolution and strengthening mechanism of Cu–Fe in-situ composites were investigated using both experiments and theoretical analysis. Experimentally, the tensile strength evolution of the in-situ composites with a cold deformation strain was studied using the model alloys Cu–11Fe, Cu–14Fe, and Cu–17Fe, and the effect of the strain on the matrix of the in-situ composites was studied using the model alloys Cu–3Fe and Cu–4.3Fe. The tensile strength was related to the microstructure and to the theoretical strengthening mechanisms. Based on these experimental data and theoretical insights, a mathematical model was established for the dependence of the tensile strength on the cold deformation strain. For low cold deformation strains, the strengthening mechanism was mainly work hardening, solid solution, and precipitation strengthening. Tensile strength can be estimated using an improved rule of mixtures. For high cold deformation strains, the strengthening mechanism was mainly filament strengthening. Tensile strength can be estimated using an improved Hall–Petch relation.

## 1. Introduction

Cu–Fe in-situ composites have a high conductivity, high strength, and low cost [1,2,3,4,5,6]. However, their strength and conductivity are decreased by the substantial solid solubility of Fe atoms in the Cu matrix at high temperatures, and the slow precipitation speed at low temperatures [7,8]. Their properties may be improved by multi-component alloying [8,9,10,11,12,13]. Wang et al. [14], Song et al. [15], and Xie et al. [16] investigated the influence of Ag. Kim et al. [17], Cui et al. [18], Song et al. [19], Jo et al. [20], Jeong et al. [21], Wu et al. [22], Chen et al. [23], and Wang et al. [24] investigated the effect of Cr, Co, Si, C, RE, M (Nb, Si, and V), and X (B, P, Si, Ge, Al, Mg, S, Cd, Ag, In, Sn, Zr, Sb, and Bi). The results indicate that the increase of tensile strength is primarily caused by second phase particles and filaments.

The microstructure of the Cu–Fe composites is composed of the Cu matrix and the Fe filaments formed during severe plastic deformation. The high strength is due to the dislocation motion hindrance of the phase interfaces [25,26]. Cold drawing causes the gradual formation of the characteristic microstructure. Increasing the cold deformation strain refines the microstructure, including the size of the second phase particles. The phase interfaces gradually become the main hindrance of dislocation motion [26,27,28,29]. The increased tensile strength is related to the filament spacing by the Hall–Petch equation [30,31,32,33,34].

(1) $$\sigma =\sigma_{0}+k\lambda^{-1/2}$$

where λ is the average filament spacing and σ_0_ is the lattice resistance of the Cu matrix. The binary Cu–Fe phase diagram shows a maximum solid solubility of 4.1% for Fe in Cu, and the diffusion rate of Fe in the Cu matrix is slow at low temperatures [9,35]. The room temperature microstructure of Cu–Fe is composed of the α-Fe phase filaments and the Cu–based solid solution. Accordingly, the strengthening of the Cu–Fe in-situ composites is attributable to (1) the phase interface strengthening caused by the Fe filaments, and (2) the matrix strengthening caused by the solid solution strengthening of the alloying elements and precipitation hardening by the second phase particles.

This paper studies the tensile strength evolution and strengthening mechanism of Cu–Fe in-situ composites using both experiments and theoretical analysis. The experimental aims were (1) to understand the strength increase with the increasing strain and (2) to establish the strengthening mechanisms. The novelty of the research is the new mathematical model for the increase of strength with increasing the cold deformation strain, based on these experiments and theoretical analysis.

The evolution of the tensile strength with cold deformation for in-situ composites was studied using the model alloys Cu–11Fe, Cu–14Fe, and Cu–17Fe, in Section 3.1, and the filament strengthening was analyzed in Section 3.2. The effect of the cold deformation stain on the matrix of the in-situ composites was studied using the model alloys Cu–3Fe and Cu–4.3Fe, in Section 3.3. The new mathematical model was established in Section 4.

## 2. Experimental Details

The model experimental alloys of Cu–xFe (wt.%; x = 3, 4.3, 11, 14, and 17) were prepared using a vacuum induction furnace with a magnesia crucible and a cylindrical graphite mold (diameter d = 36 mm). The raw materials were industrially pure Fe (99.94 wt.%) and electrolytic Cu (99.96 wt.%). The ingots were homogenized at 950 °C × 4 h, and hot rolled at 850 °C to a diameter of 25 mm. The in-situ composites were produced by cold drawing using successive drawing dies at an ambient temperature. The cumulative cold deformation strain was calculated by the logarithmic strain [7]:

(2) $$\eta =\ln\left(A_{0}∕A_{f}\right)$$

where A_f_ and A_0_ are the transverse section area of the drawn wire and of the ingot after hot rolling, respectively.

Samples were cut out from the Cu–Fe ingots and composite wires with various strains. The microstructure was characterized using a scanning electron microscope (SEM) installed with an energy dispersive spectrometer (EDS). The second phase spacing λ was obtained using two different methods. (1) For the low cold deformation strains, Fe filaments were not completely formed and the distribution of Fe grains was not uniform, the spacing between the second phase grains was calculated from [35] λ = τ·f_Cu_/f_Fe_, where f_Fe_ and f_Cu_ were the volume fraction of Fe and Cu, and τ was the average thickness of the deformed grainsm which was obtained from the transverse section SEM image. (2) For high strains, the spacing of the filaments was measured directly from the longitudinal section SEM image. The measurement accuracy of each value was 0.1 μm.

The tensile strength of the Cu–Fe alloys after different cold deformation strains was obtained at an ambient temperature using a tensile-testing machine, using custom-designed wire grips [36] for samples with high cold deformation strains. The orientation of the wire in the tensile tests was in the drawing direction. Each strength value was the average of four measurements with deviation being within 3%.

## 3. Results and Discussion

### 3.1. Cu–Fe In-Situ Composites

#### 3.1.1. Microstructures

**Figure 1:**
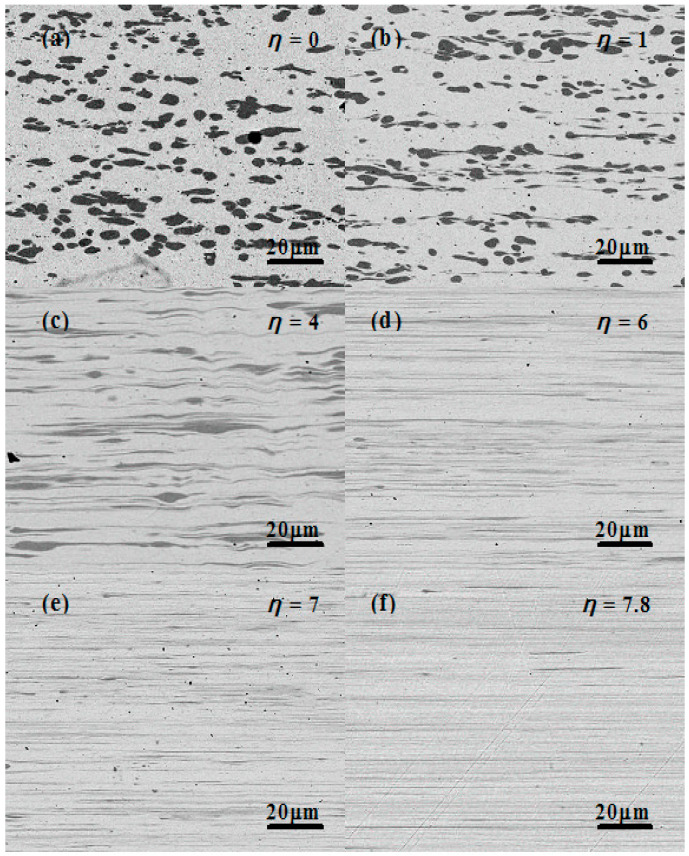
Cu–14Fe SEM microstructures at various strains: (**a**) η = 0; (**b**) η = 1; (**c**) η = 4; (**d**) η = 6; (**e**) η = 7; (**f**) η = 7.8.

Figure 1 shows the Cu–14Fe microstructures for various cold deformation strains. Figure 1a presents the longitudinal section microstructure of Cu–14Fe after hot rolling. The iron dendrite with a random orientation extended slightly parallel to the deformation direction, but the magnitude of the change was small. The Cu–14Fe after hot rolling was used as the starting material of cold deformation, that is, the cold deformation strain was η = 0. Figure 1b–f presents the evolution of the Cu–14Fe microstructure in the longitudinal section after cold deformation. When the increasing strain, the Fe dendrites were broken into grains, and some of the grains were elongated along the deformation direction, as shown in Figure 1b. When further increasing strain, the elongated Fe grains were gradually transformed into Fe filaments. At η = 4, the deformation of the Fe grains in the Cu–14Fe was not uniform; there were still some tadpole-like Fe grains, as shown in Figure 1c. When η was 6, the tadpole-like Fe grains were drawn into long thin filaments, as shown in Figure 1d. At higher strains, the deformation and distribution of the filament were more uniform. The average spacing and average size of the filaments were further decreased, as shown in Figure 1e,f.

#### 3.1.2. Tensile Strength

**Figure 2:**
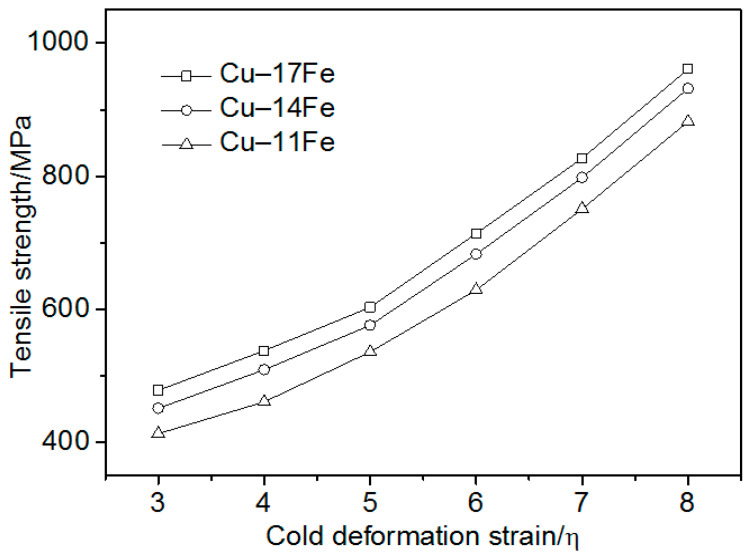
Cu–Fe strength vs. the cold deformation strain.

Figure 2 indicates that the strength of the Cu–Fe alloys increased with increasing the strain. At low strains, the increase of strength was relatively slow. For higher strains, the increment of strength increased. The filament reinforcement phase was not yet completely formed at the beginning of the cold deformation, and the strength was mainly determined by the rule of mixtures. The strengthening was due to the work hardening of the Cu matrix [13,26,35]. For higher strains, the filament reinforcement phase was gradually formed, and the strength was primarily determined by the Hall–Petch relation [8,19,35]. The uniform fine filaments arranged along the cold drawing axis effectively increased the tensile strength. The tensile strength increased with increasing the Fe content at each strain. The increment of strength for the Fe content increase from 11% to 14% was larger than that for the Fe content increase from 14% to 17%, suggesting that there is not a simple linear relationship between the tensile strength of the Cu–Fe in-situ composites and Fe content.

These observations were consistent with previous work [10,33,37], that the mechanisms by which the strains increased the strength of the composites depended on the amount of cold deformation.

### 3.2. Filament Strengthening

**Figure 3:**
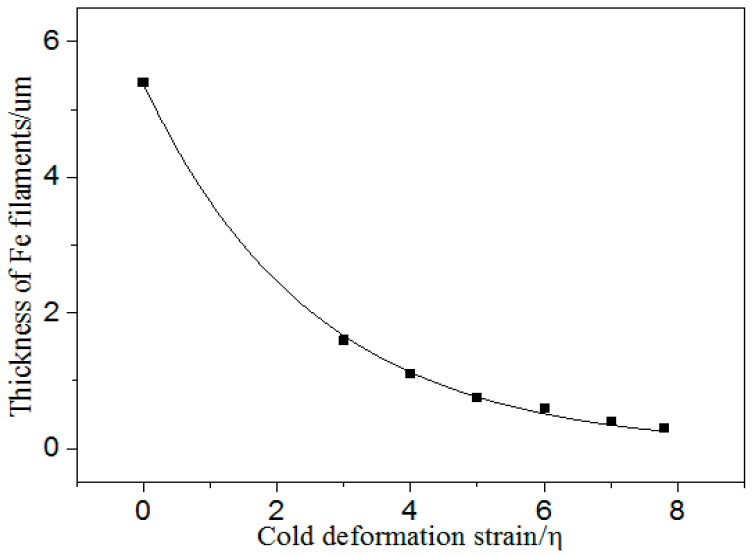
Thickness of the Fe phase in the Cu–14Fe vs. the cold deformation strain.

Figure 3 indicates that the average thickness, τ, of the Fe phase decreased with the increasing strain. Equation (3) and the constants were obtained by fitting the thickness of the Fe filaments and cold deformation strain in Figure 3 by Origin 8.5, as follows:

(3) τ=5.38exp(−0.39η)(μm)

The fitting correlation coefficient of Equation (3) was 0.999. This refers to the fitting degree of the regression equation to the observed value. The maximum value of the coefficient is 1. The closer the value of the coefficient is to 1, the better the fitting degree of the regression equation is to the observed value.

**Figure 4:**
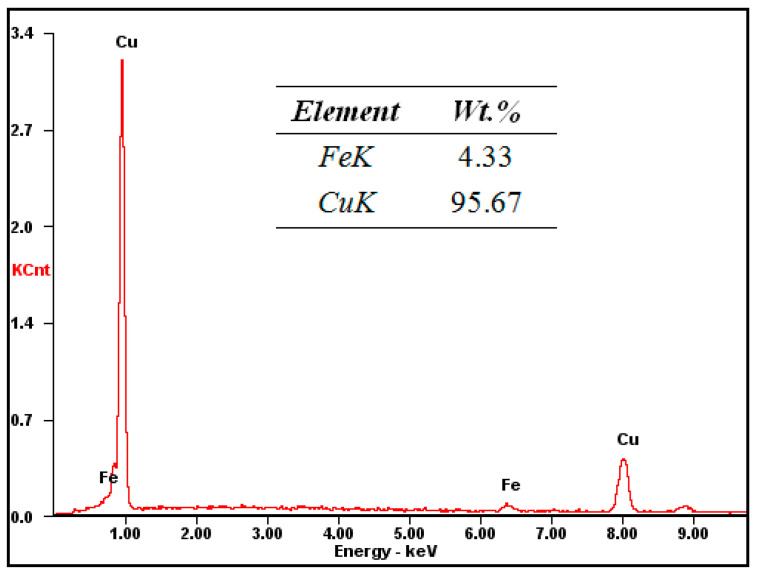
EDS analysis of the Cu–14Fe alloy.

At low cold deformation strains, the average spacing, λ, was estimated from the average thickness of the second phase, according to the following formula [38]:

(4) $$\lambda =\tau \cdot f_{Cu}/f_{\mathrm{Fe}}$$

where f_Cu_ and f_Fe_ are the volume fractions of the Cu matrix and Fe phase. f_Fe_ was determined from the content of Fe in the Cu matrix. The EDS analysis showed that the content of Fe in the Cu matrix was 4.33%, as shown in Figure 4. This exceeded the maximum equilibrium solubility of Fe in Cu. The peritectic reaction is almost impossible to complete under the experimental cooling condition, due to the fact that the peritectic temperature (1096 °C) of Cu and Fe is very close to the melting point of Cu (1084 °C), which increased the solid solubility of the solute [8]. The mass fraction of each phase was evaluated from the mass fraction of the primary Fe phase from the content of Fe in the matrix measured by EDS, then the volume fraction of each phase was evaluated through conversion from the mass fraction.

**Figure 5:**
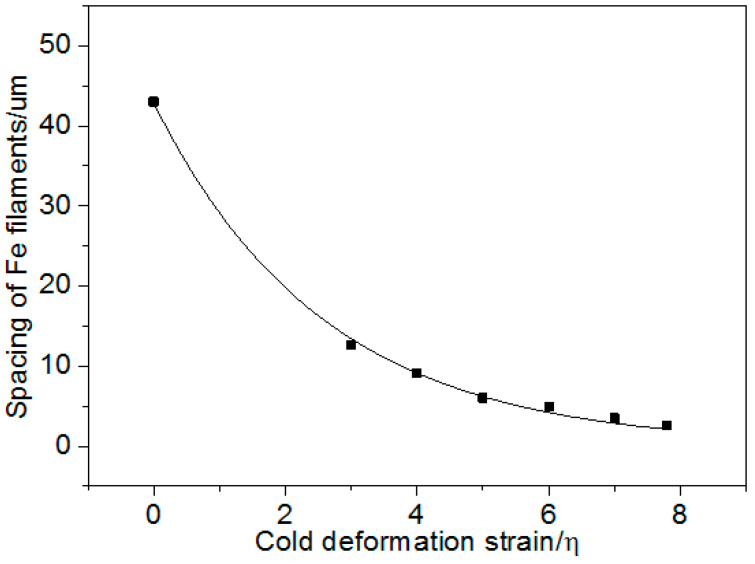
Spacing of the Fe phase in the Cu–14Fe vs. the cold deformation strain.

Figure 5 shows that the Fe phase spacing decreased gradually with increasing the cold deformation strain. Equation (5) was determined by fitting the spacing of the Fe filaments and the cold deformation strain in Figure 5 by Origin 8.5, as follows:

(5) λ=42.85exp(−0.39η)(μm)

The fitting correlation coefficient of Equation (5) was 0.998.

Equations (3) and (5) indicate that the thickness and spacing of the Fe phase both decreased exponentially with increasing the cold deformation strain. In addition, Figure 2 and Figure 5 suggest that the tensile strength increased with decreasing the second phase spacing.

### 3.3. Matrix Strengthening

**Figure 6:**
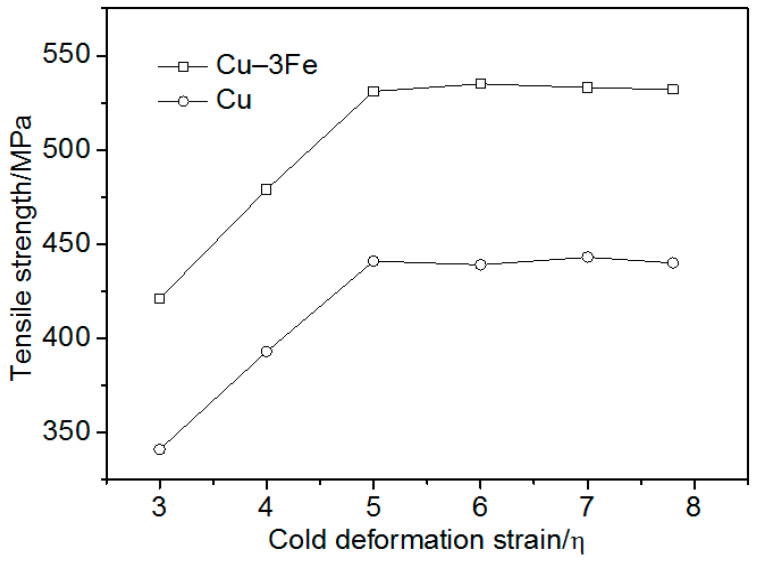
Strength of Cu and Cu–3Fe vs. the cold deformation strain.

Figure 6 presents the strength of pure Cu and Cu–3Fe for different cold deformation strains. Based on the maximum solid solubility of 4.1% for Fe in Cu and the Fe content in the Cu matrix of Cu–14Fe in Figure 4, Cu–3Fe was a single-phase Cu-based solid solution containing almost no primary Fe phase. This is consistent with previous publications [8,35]. Accordingly, Cu–3Fe was selected as the model alloy to investigate the effect of solid solution, and precipitated the Fe particles on the tensile strength of the Cu matrix.

Figure 6 shows that the pure Cu strength increased with increasing the strain at low cold deformation strains, and tended to saturation as the cold deformation strain reached 5. This was consistent with previous investigations [30,39], which showed the work hardening of face-centered cubic (f.c.c.) metals tended to saturation with increasing the strain; the pure Cu strength tended to be saturated at η = 5. Figure 6 also shows that the Cu–3Fe strength increased with increasing the strain at low cold deformation strains, and tended to saturation at η = 5. Previous works [37,40] have suggested that work hardening is closely related to dislocation behavior. The dislocation density in Cu–Fe increased with increasing the cold deformation strain. Dislocation cells were gradually formed and the dislocation cell size decreased with increasing the strain. The increasing dislocation density and the decreasing dislocation cell size made the dislocation slip more difficult, causing work hardening. As the cold deformation strain reached a certain value, the dislocation cell size decreased to a saturation value [41]. Further increasing cold deformation strain caused the movement and disappearance of dislocations and point defects, which resulted in dynamic recovery. This decreased the dislocation density. The increasing and decreasing dislocation density caused by the cold deformation strain and dynamic recovery, respectively, reached an equilibrium, and the tensile strength was saturated [33,40,42].

The strength of pure Cu and Cu–3Fe with different cold deformation strains significantly depended on the work hardening caused by the strain increase as well as the dislocation density decrease caused by the dynamic recovery. The higher tensile strength of Cu–3Fe was attributed to the addition of Fe, which existed as solid solution Fe atoms, and precipitated Fe particles in the Cu matrix to cause a solid solution and precipitation strengthening [8,35]. Accordingly, the matrix strengthening mechanism of the composites was mainly work hardening, solid solution, and precipitation strengthening. The effect of the solid solution and precipitated Fe particles on the strength of the Cu matrix could be analyzed by the strength of Cu–3Fe compared with Cu in Figure 6.

## 4. Strengthening Model

### 4.1. Rule of Mixtures

Previous research [33,39,43] has indicated that the strength of Cu-b.c.c. in-situ composites can be calculated using the rule of mixtures (ROM), as follows:

(6) $$\sigma_{C}=\sigma_{Cu}f_{Cu}+\sigma_{X}f_{X}$$

where σ_C_, σ_Cu_, and σ_X_ are the strength of composite, Cu matrix, and X phase, respectively, and f_X_ and f_Cu_ are the volume fraction of the X phase and Cu matrix, respectively.

**Figure 7:**
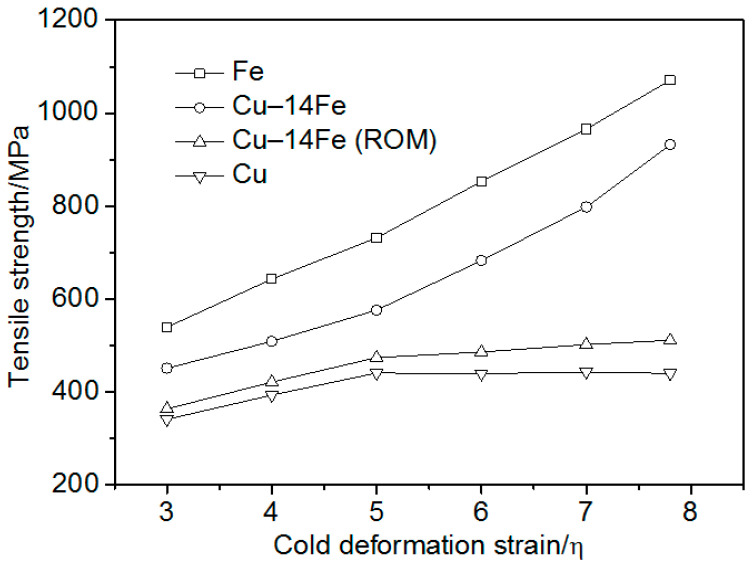
Strength of Fe, Cu–14Fe, Cu, and strength of Cu–14Fe calculated using the rule of mixtures (ROM) vs. the cold deformation stain.

Figure 7 presents the strength of Cu–14Fe, pure Cu, and pure Fe, and the strength of Cu–14Fe calculated according to the ROM. The strength of Cu–14Fe calculated based on the ROM was lower than the measured tensile strength. For cold deformation strains less than 5, the tensile strength difference was about 100 MPa. For increasing the cold deformation strain, the tensile strength difference further increased, and the ROM calculated strength of Cu–14Fe was much lower than the measured strength. The calculation used the tensile strength of pure Cu as that of the Cu matrix, σ_Cu_, which may be the main reason for the low calculated strength. Accordingly, the strength of the Cu matrix should be further discussed in order to investigate the influence of the second phase on the tensile strength of the composites, so as to build an effective strengthening model.

**Figure 8:**
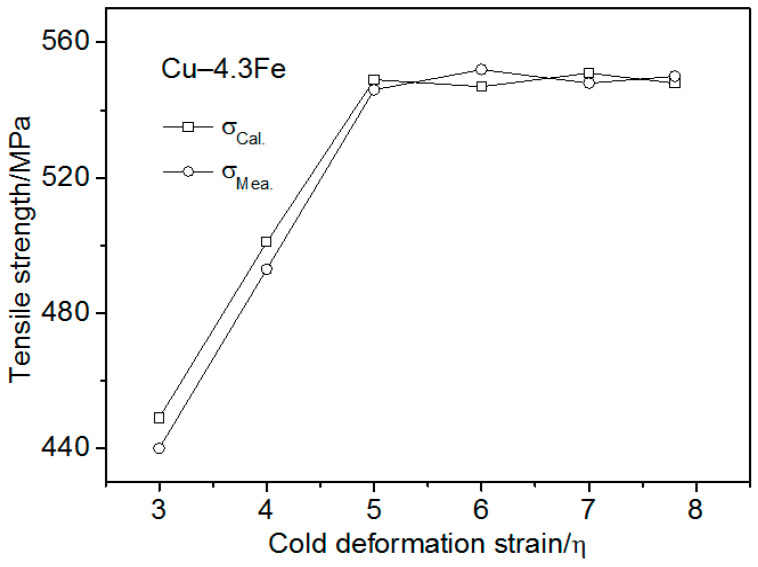
Measured and calculated tensile strength of Cu–4.3Fe vs. the cold deformation strain.

From Figure 6 and the particle strengthening mechanism mentioned above, it is indicated that there was about 90 MPa strength gap between Cu–3Fe and pure Cu, which was caused by the solid solution and precipitated Fe particles in Cu–3Fe. A previous work [44] showed that the solid solution atom group formed a quasi-precipitation particle at a relatively high solute concentration. The critical resolved shear stress, τ_c_, was given by the following expression:

(7) $$\tau_{c}=k_{1}C^{1/2}$$

where k_1_ is a constant and C is the concentration of solution atoms. The strengthening effect of the precipitated particles can be evaluated with the following expression:

(8) $$\tau_{c}=k_{2}f^{1/2}$$

where k_2_ is a constant, and f is the volume fraction of the precipitated particles. Accordingly, it can be assumed that the matrix strengthening effect, Δσ_Fe−M_, caused by the solid solution Fe atoms and the precipitated Fe particles in the Cu matrix accords with Equation (8). In addition, the volume fraction of Fe in the Cu matrix is directly proportional to the mass fraction. Equation (8) indicates that the matrix strengthening effect, Δσ_Fe−M_, can be expressed as follows:

(9) $$\Delta \sigma_{Fe-M}=k_{3}m_{Fe}^{1/2}$$

where k_3_ is a constant, and m_Fe_ is the mass fraction of the solid solution and precipitated Fe particles. The value of k_3_ is about 52, evaluated by substituting the tensile strength difference between Cu–3Fe and pure Cu into Equation (9). In order to verify the relation between the matrix strengthening effect and the mass fraction, Cu–4.3Fe was prepared. Figure 8 presents the strength of the Cu–4.3Fe as-measured and calculated using Equation (9). The measured and calculated tensile strength of the Cu–4.3Fe was the same at each cold deformation. This verified the use of Equation (9) to predict the strengthening influence of the solid solution Fe atoms and precipitated Fe particles in the Cu matrix.

**Figure 9:**
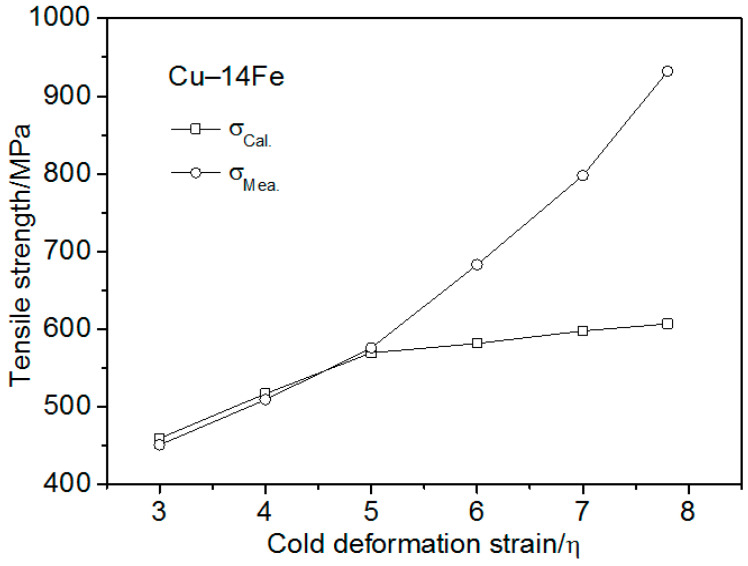
Cu–14Fe strength as-measured and calculated using the improved ROM vs. the cold deformation strain.

Figure 5 shows that the mass fraction of Fe m_Fe_ was 4.33 in the Cu matrix of the Cu–14Fe. The matrix strengthening effect Δσ_Fe−M_ was about 108 MPa, evaluated by substituting the mass fraction m_Fe_ and the constant k_3_ into Equation (9). The tensile strength of the Cu matrix, σ_Cu_, was obtained from the matrix strengthening effect, Δσ_Fe–M_, and the tensile strength of pure Cu. Accordingly, the tensile strength of Cu–14Fe, calculated using Equation (6) (Figure 9) was compared with the measured tensile strength. The strength calculated using the ROM with the improved strength of the Cu matrix was consistent with the measured strength when the cold deformation stain was less than 5. However, the calculated tensile strength was lower than the measured strength when the cold deformation strain was more than 5. The strength difference increased with the increasing strain.

The above analysis shows that the strength cannot be estimated using the improved ROM at high cold deformation strains. The matrix strengthening mechanism of Cu–Fe is mainly the work hardening, solid solution, and precipitation strengthening at low strains. The tensile strength of Cu–Fe using the improved ROM is given by the following:

(10) $$\sigma_{C}={\;\sigma }_{M}f_{M\;}+\sigma_{\mathrm{Fe}}f_{\mathrm{Fe}}$$

where σ_M_ and f_M_ are the strength and the volume fraction of the Cu matrix containing solid solution atoms and precipitated particles, and σ_Fe_ is the tensile strength of the Fe phase. The tensile strength of the Cu matrix can be calculated by the matrix strengthening effect, Δσ_Fe–M_, and the strength of the pure Cu with corresponding strain, σ_P_, as follows:

(11) $$\sigma_{M}={\;\sigma }_{P}+k_{3}m_{\mathrm{Fe}}^{1/2}$$

### 4.2. Filament Strengthening

Previous research [42,43,44,45] has indicated that the grain size of Cu-b.c.c. alloys decreases gradually with increasing the strain. The filaments form along the drawing direction when the strain reaches a certain value, and the interface between the Fe filaments and Cu matrix becomes the main strengthening mechanism to hinder the dislocation motion. The strength of the composites increases with decreasing the filament spacing. Spitzig et al. [33] found that the strength and filament spacing of Cu–Nb composites obeyed the Hall–Petch relationship of Equation (1) with σ_0_ ≈ 0 MPa, attributed to the fact that Nb is almost insoluble in the Cu matrix. In this work, the lattice resistance of the Cu matrix, σ_0_, is high because of the large solid solubility of Fe in the Cu matrix at high temperatures and the slow precipitation at low temperatures. Based on the previous analysis of the strengthening effect of the solid solution and precipitated particles on the matrix, the matrix lattice resistance of the Cu–14Fe composites is about 108 MPa, estimated by Δσ_Fe−M_. Previous research [34,35,39] has shown that the constant, k, in the Hall–Petch relation of binary Cu-b.c.c. composites is closely related to the shear modulus, G_1_ and G_2_, of each phase, as follows:

(12) $$k\approx {\left[(1+K_{c})/(1-K_{c})\right]}^{1/2}$$

where K_c_ can be represented by the following equation:

(13) $$K_{c}=(G_{2}-G_{1})/(G_{2}+G_{1})$$

In this work, the value of the constant, σ_0_, was about 108 MPa, evaluated by substituting the mass fraction, m_Fe_, and the constant, k_3_, into Equation (9); the constant, K_c_, was calculated by substituting the shear modulus of Cu 48.3 GPa and the shear modulus of Fe 81.6 GPa into Equation (13), so that the constant, k, was about 1299 MPa·µm^−1/2^, according to Equation (12). Accordingly, at high cold deformation strains, the Hall–Petch relation of the strength of the Cu–14Fe composites can be written as follows:

(14) $$\sigma_{C}=108+1299\lambda^{-1/2}$$

**Figure 10:**
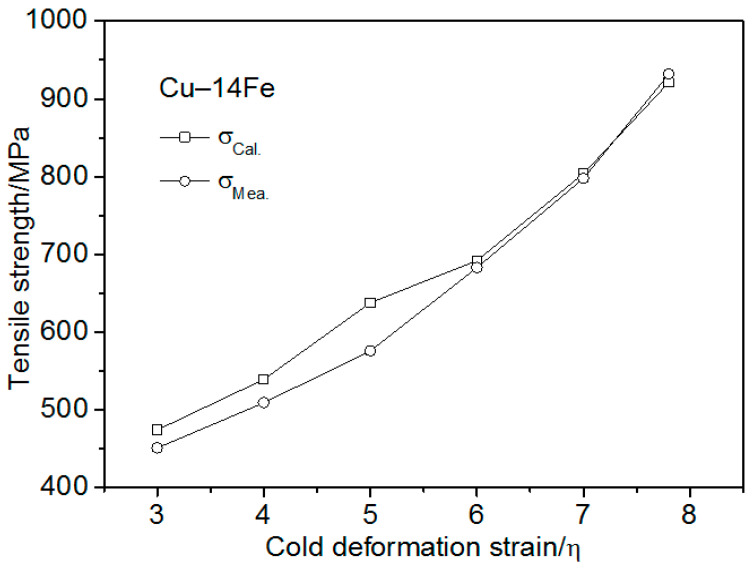
Cu–14Fe strength as-measured and calculated using the improved Hall–Petch relation vs. the cold deformation strain.

Figure 10 shows the Cu–14Fe strength as-measured and calculated using Equation (14). The tensile strength calculated using the improved Hall–Petch relation was in good agreement with the measured strength when the cold deformation strain was more than 5. However, the calculated tensile strength was higher than the measured strength when the cold deformation strain was less than 5, which indicates that the strength cannot be estimated using the improved Hall–Petch relation at low cold deformation strains.

### 4.3. Combinatorial Strengthening

The results of Section 4.1 indicate that when η is less than or equal 5, the Cu–Fe strength can be calculated using the improved ROM; the Cu matrix strength, σ_M_, can be calculated by the matrix strengthening effect, Δσ_Fe–M_, and the pure Cu strength with corresponding strain. The results of Section 4.2 indicate that when η is more than 5, the Cu–Fe strength can be calculated using the improved Hall–Petch relation. The combinatorial strengthening model is as follows:

(15) $$\{\begin{array}{cc} \sigma_{C}{=\sigma }_{M}{\;f}_{M}{+\sigma }_{\mathrm{Fe}}{\;f}_{\mathrm{Fe}} & \left(\eta \leq 5\right)\\ \sigma_{C}{=108+1299\lambda }^{-1/2} & \left(\eta >5\right) \end{array}$$

**Figure 11:**
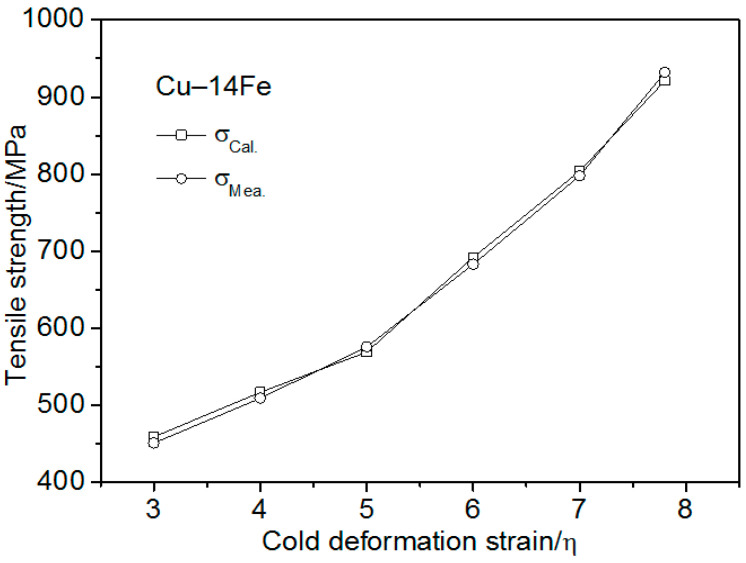
Cu–14Fe strength as-measured and calculated using the combinatorial strengthening model vs. strain.

The Cu–14Fe strength with different cold deformation strains was calculated using Equation (15) and was compared with the measured values in Figure 11. Figure 11 shows that that the tensile strength calculated using the combinatorial strengthening model was consistent with the measured strength for all of the cold deformation stains. This indicates that the Cu–Fe strength can be estimated using the combinatorial strengthening model.

## 5. Conclusions

(1) For the Cu–Fe in-situ composites, the second phase Fe dendrites with a random orientation were gradually transformed into Fe filaments, and the average filament spacing and size decreased with increasing the cold deformation strain.
(2) The Cu–Fe strength increased with increasing the cold deformation strain and with increasing the Fe content.
(3) The average spacing and size of the second phase in the composites decreased exponentially with increasing the strain.
(4) The strength of the pure Cu and single-phase Cu–based solid solution first increased and then tended to constant values with the increasing strain. The strength difference was mainly caused by the solid solution atoms and precipitated particles in the matrix.
(5) The Cu–Fe strength can be evaluated using the combinatorial strengthening model. For a low strain of η ≤ 5, the Cu–Fe strength can be estimated using the improved rule of mixtures. For a high strain of η > 5, the strength can be estimated using the improved Hall–Petch relation.
